# Uterine fibroid characteristics and sonographic pattern among Ghanaian females undergoing pelvic ultrasound scan: a study at 3-major centres

**DOI:** 10.1186/s12905-016-0288-4

**Published:** 2016-02-16

**Authors:** Benjamin Dabo Sarkodie, Benard Ohene Botwe, Eric K. Ofori

**Affiliations:** University of Ghana School of Medicine and Dentistry, P. O. Box GP 4236, Accra, Ghana; Department of Radiography, School of Biomedical & Allied Health Sciences, University of Ghana, P.O Box KB 143, Accra, Ghana

**Keywords:** Uterine fibroids, Sonographic patterns, Echo pattern

## Abstract

**Background:**

Uterine fibroids are the most common benign tumours affecting premenopausal women and are often associated with considerable hospitalization and morbidity.

The purpose of this study was to identify the uterine fibroid characteristics and sonographic patterns of uterine fibroids among Ghanaian women undergoing abdomino-pelvic or pelvic ultrasound scan at three major diagnostic centres. The outcome is expected to help in appropriate policy formulation in women care in Ghana.

**Method:**

A total of two hundred and forty four (244) women were evaluated between November 2011–February 2012, using identical 2–5 MHz curvilinear probe of Philips HD3 ultrasound machines at three major diagnostic centers in Ghana, using a trans-abdominal pelvic approach.

**Results:**

The range, mean and standard deviation (SD) of the patients’ ages were 14–54 years, 31.89 years and ± 7.92 respectively. The majority, 57.8 % of the fibroids were intramural with only 4.4 % noted as sub-mucosal. Most (55.6 %) of the fibroids were located in more than one part of the uterus. The most popular (55.6 %) echo pattern of the various fibroid nodules was mixed echogenicity.

**Conclusion:**

The sonographic patterns of uterine fibroids among Ghanaian women have been assessed at three major diagnostic centres. The study shows that most Ghanaian women who have fibroids have degenerative fibroid nodules as these nodules demonstrate mixed echo patterns on ultrasound. The findings may aid in appropriate diagnosis and interventions in the country.

## Background

Uterine leiomyoma present a major public health problem. It is the most common benign gynecologic tumor affecting premenopausal women [1, 2] which may be associated with considerable hospitalization and morbidity [3, 4]. Fibroid is considered as the most common palpable mass of the female pelvis. It is estimated to occur in 20–40 % of women during their reproductive years [5, 6] and it is believed that these tumors develop in the majority of American women and become symptomatic in one-third of these women [1, 7]. Fibroids are the most frequent indication for hysterectomy (abdominal and vaginal) and a leading cause of hospitalizations for gynecologic disorders [8, 9], accounting for approximately one third of all procedures performed annually in the United States and is suggested that black women have a greater fibroid burden than whites [10, 11]. Although the etiology of fibroids remains unknown, the female hormones estrogen and progesterone are hypothesized to enhance fibroid [12, 13]. The exact data on prevalence of fibroid condition are lacking, primarily because of limited population-based studies, varying symptomatology [14] and differences in case definitions across studies. A population-based study [15] in the United States found a cumulative incidence of uterine fibroids of greater than 66 % by ultrasound examination of women approaching age 50 years. It has been suggested that the natural history of fibroids is poorly understood, which makes it difficult to advise asymptomatic women with fibroids on the risk of developing clinical symptoms in the future [4].

The use of ultrasound to diagnose and monitor the growth of fibroid [7] has been well accepted. Fibroids appear on ultrasound in various echo patterns namely hypo-echoic, iso-echoic, hyper-echoic and mixed echo pattern [7]. Uncomplicated fibroids are mostly hypo-echoic, but can be iso-echoic or hyper-echoic compared to normal myometrium while calcification which could be asociated with degenerated fibriod is seen as echogenic foci with shadowing^7^. However, cystic areas of necrosis or degeneration may be seen in a portion of fibroid as anechoic with poterior enhancement and or internal echogenicity [7]. According to Samir and Mohammed [16] hypoechoic pattern of fibroid nodules were the most common (79 %) echopattern noted in diagnosed fibroid nodules using sonography and followed by mixed (heterogeneous) echo texture (14.3 %).

However, many studies [15–17] on ultrasonography pattern of fibroids have been focused on symptomatic white women, with limited study on African women or women with asymptomatic disease.

In Ghana, information on the ultrasonography pattern of fibroids is essential for policy formulation. The only study by Ofori et al. [18] have tried to assess the sonographic pattern of fibroid of Ghanaian women using ultrasound and found most of the women to have hypoechoic patterns. However, it was a single site study, and the study considered only fibroid among women above 20 years, neglecting those below 20 years. It is against this backdrop that this study was undertaken to vigorously establish the sonographic pattern and uterine fibroid characteristics of uterine fibroid among Ghanaian women. The intention was to identify the sonographic patterns among women undergoing trans-abdominal ultrasonography suspected to have uterine fibroids, with the view that, findings could aid in appropriate policy formulation in women care in Ghana.

## Methods

A quantitative prospective cross-sectional quasi-experimental study design was used to evaluate the uterine fibroid characteristics in two hundred and forty four (244) women of childbearing age by trans abdominal pelvic ultrasound scan. The study took place in three major diagnostic centres in Ghana. The centres were chosen because they had identical scanning equipment models and large patient turn over. The study was conducted between November 2011–February 2012. A month was allocated for each diagnostic centre and all patients who were referred to these three major diagnostic centres between the study period, to undertake abdominal-pelvic or pelvic ultrasound examinations were informed and recruited for the study. These patients included females who were 15 years old and over; however, excluded were patients with history of hysterectomy. In the study site, people below 18 years are considered minors. Therefore parental consent was obtained for those below 18 years. Consent was also obtained from all the adult participants. In all, 81, 99 and 64 patients were recruited in centres 1, 2 and 3. To ensure quality control, all the scans were done by one specialist radiologist with 10 years experience in obstetrics scanning. To obtain data, all patients were placed supine on the examination couch. Coupling gel was applied to the suprapubic region after exposure, and systematic scanning commenced by moving the transducer (identical curvilinear probe of Philips HD3 with frequencies between 2 MHz and 5.0 MHz) over this region. All pelvic ultrasound images generated at the centres within the study period were evaluated and analysed. The data collected was collated and analysed using the Statistical Package for Social Sciences (SPSS) version 17.0 for windows, 2009; Chicago. Descriptive statistics such as means, standard deviations, frequencies, percentages and proportions were calculated. Chi-square was used to assess age of respondents and their fibroid characteristics. Data was also summarized using tables, bar charts, pie and charts. All tests were two-tailed and p-value of less than 0.05 was interpreted as significant.

### Ethical consideration

In accordance with ethical requirement, ethical clearance was first sought and obtained from the Ethics committee of Ghana Medical School, College of Health Sciences, University of Ghana. Approval was also sought from the Medical Directorate of the selected centres. Because cooperation and commitment of the partners involved in the project are crucial, discussions were held with them prior to the study. This decision was taken in order to get (i) their individual consent and (ii) to encourage them to participate voluntarily and encourage co-operation. Since additional data other than that provided on patients’ request forms was recorded, patients were made aware of the importance of the study for which data was being taken and the need for their cooperation and consent. They were also informed that the data being taken would not in anyway, affect the results of their examination to be done. Additionally, they were assured of confidentiality of their identity and the information collected on them. Finally, patients signed a consent form signifying that they had agreed to the use of the data collected for the study for research purposes.

### Machine/Technique and protocol

Ultrasound scanning machines at the selected sites were used in the study. Table 1 shows the characteristics of Ultrasound scanners available in the centres. All three centres had identical equipment specifications.

**Table 1:** Ultrasound parameters of the equipment used

Parameters of the equipment	Details
Type	HD
Manufacturer	PHILIPS MEDICAL
Model	HD3
Year of manufacture	2005
Probe	Curvilinear
Frequency of probe	2–5 MHz

Technical specifications of the ultrasound scanning equipment used in all three (3) centres are shown in Table 1.

## Results

**Fig. 1:**
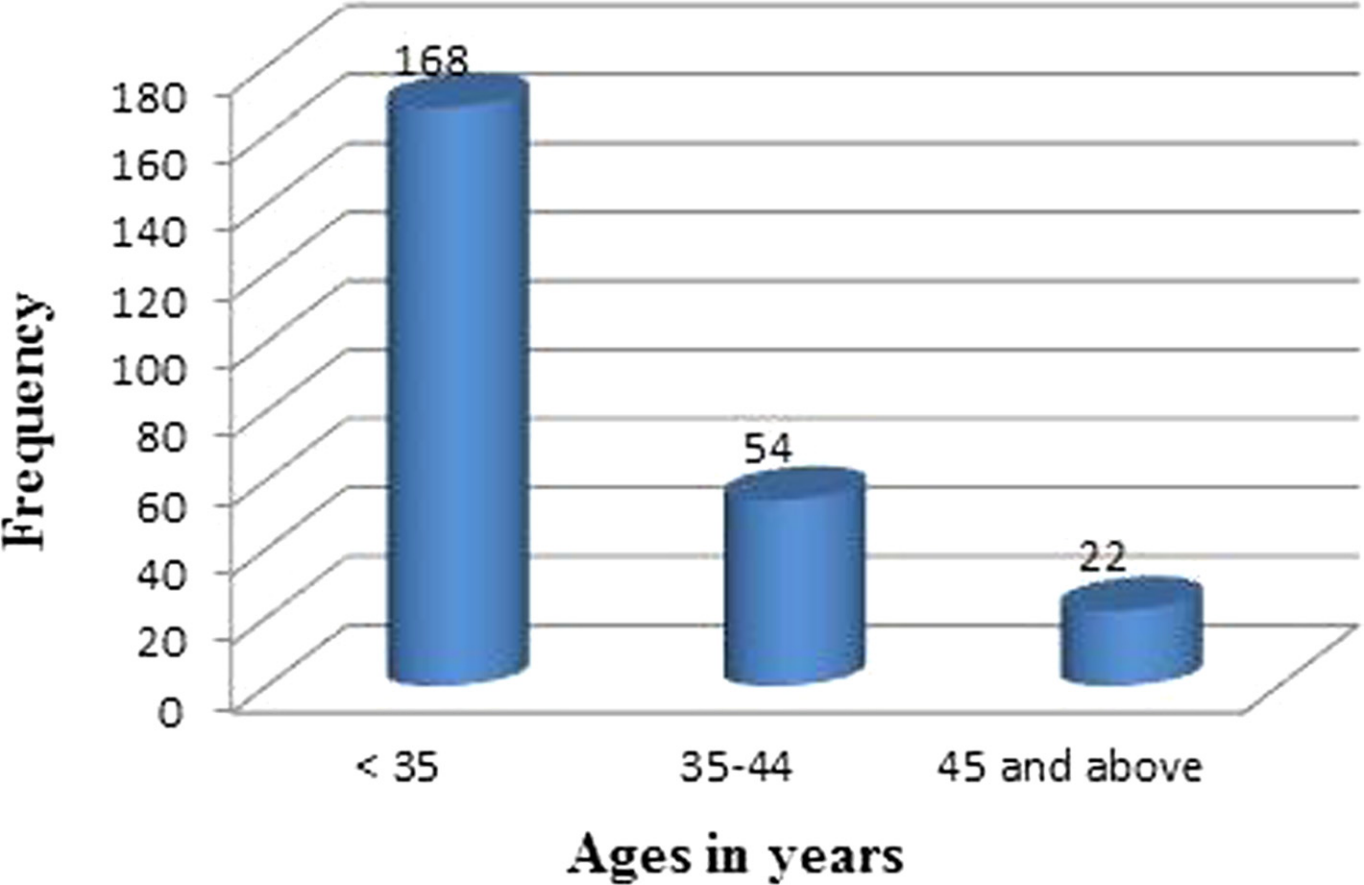
Age distribution of participants

Two-hundred and forty-four (244) patients were evaluated by this study. The age range, mean and standard deviation (SD) of patients were 14–54 years, 31.9 years and ± 7.9 respectively. The age distributions of participants are shown in Fig. 1.

Prior to the study, 35.2 % (86/244) of the participants stated that they had been previously diagnosed to have fibroids. However, during the study, only 71.1 % (64/86) of them were confirmed of having fibroids. Additionally, out of the 158 patients who did not know previously of having fibroid, 26 representing 28.9 % were confirmed to have fibroid. In general the prevalence or confirmed rate of the participants having fibroid was 36.9 % (90/244).

Table 2 illustrates cross tabulation of participant’s previous diagnosis of fibroids prior to the study against participants confirmed diagnosis of fibroids during the study.

**Table 2:** Confirmation of participants who have fibroid from the study

		Confirmed diagnosis of fibroids in participants		Total
		Yes	No	
Previous diagnosis of participants having fibroids	Yes	64	22	86
		71.1 %	14.3 %	35.2 %
	No	26	132	158
		28.9 %	85.7 %	64.8 %
Total		90	154	244
		100.0 %	100.0 %	100.0 %

The diameter of the largest fibroid nodules were measured and categorised. The study revealed that sizes of the nodules were evenly distributed (Table 3). The average number of fibroid nodules and diameter were estimated as, 3.3 and 2.7 cm respectively. Table 4 shows that fibroid sizes and age ranges of participants. It is observed that fibroid sizes less than 3 cm were 47.4, 22.3 and 25.0 % prevalence among women below 35 years, 35–44 years, and 45 and above years respectively. Fibroid sizes between 3 and 4.9 cm were also 26.3 % prevalent in women below 35 years, 33.3 % in women between 35 and 55 years, and 37.5 % in women at 45 and above years. In addition, fibroid sizes 5 cm and above were found in 26.3, 44.4 and 37.5 % of women aged under 35 years, 35–44 years, and 45 and above years respectively.

**Table 3:** Sizes of fibroid nodules

Diameter of the largest fibroid nodule	Frequency	Percentage
<3 cm	30	33.3
3–4.9 cm	28	31.1
5 cm and above	32	35.6
Total	90	100.0

**Table 4:** Age of participants and fibroid sizes

		Age Range of patients in years			Total
		<35	35–44	45 and above	
Fibroid sizes	<3 cm	18	8	4	30
		47.4 %	22.2 %	25.0 %	33.3 %
	3–4.9 cm	10	12	6	28
		26.3 %	33.3 %	37.5 %	31.1 %
	5 cm and above	10	16	6	32
		26.3 %	44.4 %	37.5 %	35.6 %
Total		38	36	16	90
		100.0 %	100.0 %	100.0 %	100.0 %

The sonographic patterns of uterine fibroids in terms of classification and location in the uterus are represented in Tables 5, 6 and 7. The findings in Table 5 show that intramural fibroids were predominant (57.8 %) among the sampled population. In terms of the location of fibroids in the uterus, majority (55.6 %) were classified as mixed (Table 6). Mixed echo pattern were also the commonest sonographic patterns (Table 7). A test of association between age of respondents and fibroid nodules size revealed a significant correlation between age and diameter of largest nodules (*r* = 0.209; *p* = 0.012) and number of nodules (*r* = 0.208; *p* = 0.012). Age also correlated significantly with the location of fibroid (*p* = 0.050) and sonographic pattern (*p* = 0.009).

**Table 5:** Distribution of fibroid characteristics and age of participants

	<35	35–44	45 and above	Total
	N (%)	N (%)	N (%)	N (%)
Fibroid classification				
Intramural	24 (63.2)	20 (55.6)	8 (50.0)	52 (57.8)
Subserosal	4 (10.5)	0 (0)	2 (12.5)	6 (6.7)
Pendunculated	0 (0)	2 (5.6)	2 (12.5)	4 (4.4)
Mixed	6 (15.8)	14 (38.9)	4 (25.0)	24 (26.7)
Submucosal	4 (10.5)	0 (0)	0 (0)	4 (4.4)
Total	38 (42.2)	36 (40.0)	16 (17.8)	90 (100.0)

**Table 6:** Distribution of fibroid location and age of participants

Location of fibroid	<35	35–44	45 and above	Total
Fundus	6 (15.8)	6 (16.7)	6 (37.5)	18 (20.0)
Corpus	14 (36.8)	4 (11.1)	4 (25.0)	22 (24.4)
Lower segment	0 (0.0)	0 (0.0)	0 (0.0)	0 (0.0)
Mixed	18 (47.4)	26 (72.2)	6 (37.5)	50 (55.6)
Total	38 (42.2)	36 (40.0)	16 (17.8)	90 (100.0)

**Table 7:** Distribution of echo pattern of fibroids and age of participants

Echo pattern of fibroid	<35	35–44	45 and above	Total
Hypo	6 (15.8)	6 (16.7)	6 (37.5)	18 (20.0)
Iso	14 (36.8)	4 (11.1)	4 (25.0)	22 (24.4)
Hyper	0 (0.0) 6	0 (0.0)	0 (0.0)	0 (0.0)
Mixed	18 (47.4)	26 (72.2)	6 (37.5)	50 (55.6)
Total	38 (42.2)	36 (40.0)	16 (17.8)	90 (100.0)

## Discussion

### Demographics

A total of 244 trans-abdominal pelvic ultrasound scan images of women were analysed. The age range, mean and standard deviation (SD) of patients were 14–54 years, 31.9 years and ± 7.9 respectively. Prior to the study, 35.2 % of the sampled participant’s stated that they had been previously diagnosed to have fibroids. However, during the study, only 71.1 % of that number were confirmed of having fibroids. It would have been interesting to know where and how the previous diagnosis was made, however, this information was not solicited. However, it is suggested that two factors influence the ultrasound confirmation rate of fibroid. That is, the diagnostic competence of the referring physician and the sonographer/radiologists. In our study, a specialist radiologist with 10 years experience undertook the scans post requisitions; therefore our findings are very credible. Additionally, out of the 158 patients who did not know previously of having fibroid, 28.9 % were confirmed to have fibroid. In general the prevalence or confirmed rate of the participants having fibroid was 36.9 %. The finding is consistent with available data [19] which suggest that fibroids are the most common pelvic tumour in women, causing symptoms in approximately 25 % of reproductive age women with the overall prevalence of fibroids increasing to over 70 %.

The present study indicated that the highest prevalence (42.2 %) of the fibroid cases was found among women aged <35 years and the lowest (17.8 %) recorded among women aged greater than 45 years. The current study also showed that 40.0 % women aged between 35 and 44 years also recorded significant cases of fibroid which is in agreement with previous work conducted elsewhere [19, 20]. This suggests that uterine fibroid detection by sonography is very common among women in their late 30’s and 40’s and usually shrink after menopause. Hormonal changes could account for this finding. During menopause fibroids shrink possibly due to the absence of estrogen. The findings of this study however, were in contrast with that of Lurie et al., [21] which estimated the prevalence of uterine fibroid as 4 % in women aged 20–30 years, 11 to 18 % in women between 30 and 40 years and 33 % in women between 40 and 60 years. Having fibroids at a much younger age may be related to a strong family history and the increased risk of uterine leiomyoma in people of African descent. In the lower age group, this may result in infertility and problems with childbearing. The recorded situation in Ghana is worrying in view of health and other risk implications. Fibroid has social, economic and medical implications in the female populace. Premenopausal women (18–45 years) in Ghana constitute about 40 % of the Ghanaian population [22] and are strong component of the country’s workforce and thus contribute immensely to the economy.

### Fibroid characteristics

The average number of fibroid nodules and diameter of the largest nodule were estimated as, 3.4 and 4.3 cm respectively. In women of child bearing age this may be associated with frequent miscarriages and may also be a cause of dysmenorrhea and menorrhagia. Significant correlation was found between age and diameter of largest nodules (*r* = 0.209; *p* = 0.012) and number of nodules (*r* = 0.208; *p* = 0.012). Age also correlated significantly with the location of fibroid (*p* = 0.050) and sonographic pattern (*p* = 0.009). The results agree with findings by Evans and Brunsel [23] who observed that uterine fibroid increases with age.

This lends credence to the fact that fibroids are responsive to the reproductive hormones estrogen and progesterone [24]. In premenopausal women, larger fibroid sizes are expected. Growing evidence [5] suggest that fibroids grow from tiny uterine muscle cells and may be initially diagnosed by an imaging procedure as small nodules and can grow to larger sizes where they are palpated through the abdominal wall with size ranging from 2 to 7.5 cm. The average number (3.3) of nodules recorded in the present study is lower than previously reported [25].

The results of the present study also indicated that (57.8) %) of the fibroids were intramural with only 4.4 % been submuscosal. Fibroids are muscular in origin which therefore follows that they may be more intramural leiomyoma compared to the rest. Most (55.6 %) of the fibroids were diffusely distributed within the uterus with only 20.0. % located at the fundus of the uterus. The corpus was the commonest site for location of intramural fibroids and corroborates the results of earlier studies [19]. Intra-mural fibroids located entirely within the uterine wall are noted to be the most common variety [6]. This was a consistent finding in the current study. Sub-mucosal fibroids was the least recorded (4.4 %) and was located beneath the mucosa or the endometrial lining of the uterus which is indirectly adjacent to the uterine cavity and are often clinically noted [26, 27] as the greatest cause of irregular bleeding and poor reproductive outcomes due to their closeness to the endometrium. Sub-serosal fibroids recorded the second highest frequency (25.9 %) location. They lie beneath the serosa as they are located at the outer layer of the uterus and tend to distort the outer contour of the uterus. The pedunculated type which recorded 7.7 % of all the fibroid cases was noted to be attached to the uterus by a stalk and usually grew bigger into the abdomen [26].

### Sonographic pattern

With respect to echogenicity, mixed echoic pattern of fibroid nodules was the most common recorded fibroid (55.6 %). The findings may suggest late diagnosis with degeneration which is as a result of long term presence of fibroid in these patients. This finding was in aberrant with a previous study [28] which found hypo echoic pattern as the most common (79 %) echo pattern noted in diagnosed fibroid nodules using sonography. It is also in contradiction to a previous study conducted in Ghana by Ofori et al. [18]. It observed that the difference in the findings could be that the current study did not limit the females to those above 20 years as done by Ofori et al. [18]. Broadening the age bracket to females of 15 years and above and using sample from three major locations in the country could have accounted for the observed variation.

## Conclusion

Ultrasound examination of uterine fibroid among Ghanaian females in three major diagnostic centers indicated varying sonographic patterns however, the most popular echo pattern of the various fibroid nodules was mixed echogenicity and this pattern was predominant in females under 35 years and above.

Fibroid in females at 45 years and above however, produced both mixed and hypo echogenicity.

The majority of the fibroids observed among Ghanaian females were intramural with only 4.4 % noted as sub-mucosal. Most of the fibroids were located in more than one part of the uterus. Fibroids create child bearingand marital problems relating to intercourse due to frequent and excessive bleeding, blood changes associated with menstrual cramps and heavy bleeds and therefore a health condition that requires attention.

Adequate knowledge of the sonographic patterns of fibroids among clinicians/sonographers in Ghana in order to improve the quality of diagnosis and the need of implementing appropriate quality measures that, will contribute to the provision of high-quality care in the country.

## References

[1] Saunders M, Budden A, Maclver F (2005). Dose implications of fluoroscopy-guided positioning (FGP) for lumbar spine examinations prior to acquiring plain film radiographs. Bri Med J.

[2] Engel-Hills P (2006). Radiation protection in medical imaging. Radiography.

[3] International Commission on Radiological Protection (ICRP). Publication 60 (1991). Recommendations of the International Commission on Radiological Protection. Ann ICRP.

[4] Critchley HOD, Warner P, Lee A, Brechin A, Guise J, Graham B (2004). Evaluation of abnormal uterine bleeding: comparison of three outpatient procedures within cohorts defined by age and menopausal status. HTA Monograph.

[5] Hatasaka H (2005). Evaluation of abnormal uterine bleeding. Clin Obstet Gynaecol.

[6] Goodwin SC, Spices JB, Worthington-Kirsch R, Peterson E, Prong LS, Myers ER (2008). Uterine artery embplization for treatment of leiomyomata: long term out comes from fibroid registry. Obset Gynecol.

[7] Sagba F. Uterine Leiomyoma: Urogenital, Obstetrics and Gynecology. Tag. Uterus. 2010. Available at: www.Radiopaedia.Org. [Accessed 4th May 2011].

[8] Levy B, Mukhejee T, Hirschhorn K (2000). Molecular cytogenetic analysis of uterine leiomyomas and leimyosarcoma by comparative genomic hybridization. Cancer Cytogenet.

[9] Viswanathan M, Hartmann K, McKoy N (2007). Management of uterine fibroids: an update of evidence. Agency for Healthcare Research and Quality (AHRQ): Summary, Evidence Report/Technology Assessment.

[10] Hodge JC, Quade BJ, Rubin MA, Stewart EA, Dal Cin P, Cynthia C (2008). Morton molecular and cytogenetic characterization of plexiform leiomyomata provide further evidence for genetic heterogeneity underlying uterine fibroids. Am J Pathol.

[11] Northington GM, Arya LA (2006). Uterine leiomyoma. Obstetrics and gynecology. Board Review Manual. Obstet Gynecol.

[12] Flake GP, Andersen J, Dixon D (2003). Etiology and pathogenesis of uterine leiomyomas: a review. Environ Health Perspect.

[13] Fiore K (2011). Fibroid surgery may up birth rates after recurrent miscarriage.

[14] Wilcox LS, Koonin LM, Polras R (1994). Hysterectomy in the United States, 1988–1990. Obstet Gynecol J.

[15] Velebil P, Wingo PA, Xia Z (1995). Rate of hospitalization for gynecologic disorders among reproductive-age women in the United States. Obstet Gynecol.

[16] Samir FAA, Mohammed KA. Behavior of leiomyoma durring pragnancy as evalauted by ultrasound. 2011. Available at: www.obgyn.net/displayarticle.asp. [Accessed 12th Dec 2012].

[17] Chao-Ru C, Germaine MB, Norman GC, Kimberly MP, Jean W-W (2001). Risk factors for uterine fibroids among women undergoing tubal sterilization. Am J Epidemiol.

[18] Ofori EK, Antwi WK, Arthur EA, Brakohiapa EK, Sarkodie BD, Dzefi-Tettey K, Obeng H, Adjei PK, Coleman J (2012). Prevalence and sonographic patterns of uterine fibroid among Ghanaian women (uterine fibroid- the Ghanaian situation). J Med Appl Biosci.

[19] Goodwin SC, Spies JB (2009). Uterine fibroid embolization. N Engl J Med.

[20] Jacobson GF, Shaber RE, Hung YY (2007). Changes in rates of hysterectomy and uterine conserving procedures for treatment of uterine leiomyoma. Am J Obstet Gynecol.

[21] Lurie S, Piper I, Woliovitch I, GleZeman M (2005). Age related prevalence of sonographically confirmed uterine myoma. J Obstet Gynaecol.

[22] Masters C. Are hysterectomies too common? 2007. Available at: [http://www.time.com/time/health/article/0,8599,1644050,00.html?cnn=yes. [Accessed 4th Jul 2012].

[23] Evans P, Brunsel S (2007). Uterine fibroid tumors: diagnosis and treatment. Am Fam Physician.

[24] Broder MS, Kanouse DE, Mittman BS, Bernstein SJ (2000). The appropriateness ofrecommendations for hysterectomy. Obstet Gynecol.

[25] Boulware LE, Marinopoulos S, Phillips KA, Hwang CW, Maynor K, Merenstein D, Wilson RF, Barnes GJ, Bass EB, Powe NR, Daumit GL (2007). Systematic review: the value of the periodic health evaluation. Ann Intern Med.

[26] Han PK (1997). Historical changes in the objectives of the periodic health examination. Ann Intern Med.

[27] Marshall LM, Spiegelman D, Manson JE (1998). Risk of uterine leiomyomata among premenopausal women in relation to body size and cigarette smoking. Epidemiology.

[28] Hai-Yun W, Yang L-L, Zhou S (2010). Impact of periodic health examination on surgical treatment foruterine fibroids in Beijing: a case control study. BMC Health Serv Res..

